# Effects of Mindfulness Meditation Duration and Type on Well-being: an Online Dose-Ranging Randomized Controlled Trial

**DOI:** 10.1007/s12671-023-02119-2

**Published:** 2023-04-12

**Authors:** Guy W. Fincham, Ken Mavor, Barbara Dritschel

**Affiliations:** 1 School of Psychology & Neuroscience, University of St Andrews, St Andrews, Scotland; 2 School of Psychology, University of Sussex, Brighton, England

**Keywords:** Mindfulness meditation, Dose, Technique, Component analysis, Well-being

## Abstract

**Objectives:**

This multi-arm randomized controlled online trial explored the effects of two key mindfulness characteristics (dose and type) over 2 weeks on mental well-being, along with psychological distress and dispositional mindfulness, in a healthy community sample.

**Method:**

Participants were randomly assigned to one of four mindfulness interventions (~ 10 min or ~ 30 min of sitting or movement meditation) to practice daily for 2 weeks; 161 participants fully completed the study and were included in the final sample. We also explored self-reported adherence through how often participants practiced, along with dropout rate via how many participants fully completed the study.

**Results:**

Well-being and mindfulness scores increased—and distress scores decreased—within all four conditions. However, most importantly, there were no significant differences between the conditions as a function of meditation dose or type. There were also no differences between the conditions on how regularly the meditations were practiced irrespective of type or dose. Additionally, there was no difference on dropout rate regarding meditation dose. However, meditation type had an effect, with a significantly higher dropout rate for participants allocated to a movement meditation irrespective of the dose.

**Conclusions:**

Brief mindfulness meditation may offer some benefit to well-being regardless of the meditation type and dose but, fundamentally, no differences in effects were detected between short/long sitting meditations and short/long movement meditations. Moreover, the results indicate that movement meditations may possibly be harder to adhere to, potentially informing the tailoring of mindfulness-based self-help programs. Limitations and future directions are also discussed.

**Preregistration:**

This study was retrospectively registered with the Australian New Zealand Clinical Trials Registry (ACTRN12619000422123).

**Supplementary Information:**

The online version contains supplementary material available at 10.1007/s12671-023-02119-2.

There has been a significant increase in research on meditation in the past few decades (Goleman & Davidson, 2017). Meditation encompasses numerous mental practices (Williams & Kabat-Zinn, 2013). The most widely used type of meditation across psychotherapeutic interventions is mindfulness, a technique in which one aims to approach their present-moment experience with non-reactive, unprejudiced attention (Bishop et al., 2004). Common mindfulness practices include focusing awareness on the breath and/or bodily sensations with a sense of equanimity, recognizing when the mind has wandered, and bringing the attention back to the breath or body. A highly cited systematic review and meta-analysis of 47 randomized controlled trials (RCTs) comprising of active control conditions to account for placebo effects examined meditations’ efficacy across more than 3500 participants (Goyal et al., 2014). It found that there was inadequate evidence for any impact of meditative courses on health and well-being factors such as improved sleep, weight, cognition, and mood. However, mindfulness meditation moderately reduced anxiety, pain, and depression and, to a lesser extent, improved mental well-being and reduced psychological stress (Goyal et al., 2014). There is evidence that these interventions can improve mental health in clinical (Goldberg et al., 2018) and non-clinical settings (Khoury et al., 2015), along with physical health (Creswell et al., 2019).

Furthermore, mindfulness meditation can be effective when delivered remotely (Gu et al., 2018; Spijkerman et al., 2016). This is important as online delivery of mindfulness meditations may provide time- and cost-effective solutions for the unprecedented surge in anxiety, depression, and stress levels due to events like COVID-19 which limit in-person interaction: these levels have surpassed population norms and therefore cannot be addressed through conventional, individualized mental health treatment alone (Holmes et al., 2020; Jia et al., 2020). A meta-analysis of 83 RCTs found unguided mindfulness-based self-help (MBSH) showed small, statistically significant effects at post-intervention for outcomes of well-being, stress, mindfulness, anxiety, and depression, providing evidence for such accessible interventions in public health settings (Taylor et al., 2021). Indeed, the effectiveness of fully automated mindfulness interventions has been proven (Davis & Zautra, 2013; Mak et al., 2015; Morledge et al., 2013) and full automation of treatment is needed to help meet ever-growing mental health needs.

Lower psychological stress and greater dispositional mindfulness are connected to greater mental well-being. Stress and well-being tend to have an inverse relationship, especially when there is a mismatch between an individuals’ environmental demands and coping capacity in which case stress and well-being would be higher and lower, respectively (Hogan et al., 2015). Conversely, greater levels of dispositional mindfulness have been shown to be correlated with higher well-being (Carlson & Brown, 2005).

One particular mindfulness-based program that can improve mental health and has tentative evidence supporting its feasibility and acceptability when delivered online is Mindfulness-Based Cognitive Therapy (MBCT) (Moulton-Perkins et al., 2022). MBCT is an 8-week course that involves several different components of mindfulness meditations (S1 Appendix 1)—for instance, the “Body Scan” (wherein one lies down passing attention through different parts of the body) and “Stretch and Breathe” (a combination of the sitting meditation and mindful movement practice used in this study). Two of the many core practices such as sitting meditation and mindful movement involve observing the breath and/or bodily sensations and using them as an anchor point or object for present-moment awareness. A recent systematic review and meta-analysis showed that mindfulness-based programs, including MBCT, may be connected to beneficial effects on well-being, general health, and quality of life, thus possibly offering effective treatment to non-clinical samples with subclinical levels of poor mood, stress, and anxiety (Galante et al., 2021; Querstret et al., 2020).

Although there is considerable evidence that online mindfulness/MBSH may be effective, less is known about how various meditation characteristics (such as dose and type) impact well-being. Initial findings indicate that various techniques could work on different psychological processes (Feruglio et al., 2021; Kropp & Sedlmeier, 2019) and preliminary component analyses’ results suggest mindful movement could lead to larger improvements in mental well-being than static sitting or lying mindfulness practices (Galante et al., 2021; Sauer-Zavala et al., 2013). There is a commonly held belief that lower durations are easier to practice more regularly and has greater adherence. Indeed, shorter durations have been suggested to facilitate more regular practice of mindfulness techniques (Banerjee et al., 2017). However, there is no clear evidence in the literature to suggest that the type of mindfulness meditation influences whether people will adhere more to a specific practice. Akin to physical exercise, the mental practice of mindfulness may result in augmented benefits from longer doses of practice (Goleman & Davidson, 2017). For instance, levels of the stress hormone cortisol may be modulated by meditation in this way (Fan et al., 2014). However, some studies have shown no relationship between dose of mindfulness and meditation practice and improvement on self-reported stress (Prasad et al., 2011; Strohmaier, 2020).

Thus, this online trial attempted to test two key mindfulness practice characteristics (dose [~ 8–10 min vs. ~ 29–30 min] and type [sitting vs. movement]) over 2 weeks, in a healthy community sample. The primary outcome was self-reported mental well-being, and secondary outcomes included self-reported psychological distress and dispositional mindfulness (or lack of mindlessness). Moreover, as in the case for exercise vis-à-vis augmenting physical fitness, consistent meditation practice may be needed for improvements in mental well-being (Goleman & Davidson, 2017). Hence, exploring whether a certain dose or type of meditation is subjectively easier to practice or adhere to may provide valuable data.

Our study was focused on automated MBSH. Given the strong body of evidence behind mindfulness meditation’s benefits for well-being and the fact that there are different meditation techniques of differing durations, we hypothesized that (1) all the mindfulness meditation interventions will be associated with an increase in mental well-being scores (our primary outcome) and the mindfulness measure (secondary outcome), and a decrease in psychological distress (secondary outcome); however, the magnitude of these changes may vary according to the type and/or dose of meditation. For instance, longer doses of meditation should theoretically lead to greater increases in well-being. Yet, such longer duration meditations may be practiced less than shorter duration meditations, and there could be differences across conditions as a function of self-reported adherence. Again, shorter doses are facilitators and longer doses are barriers to meditation practice, respectively (Banerjee et al., 2017). Accordingly, such meditation dose could also influence whether participants completed the study or dropped out. In line with the above, and based on previous literature, we hypothesized that (2) the shorter mindfulness meditations would be practiced more regularly than the longer mindfulness meditations and greater self-reported adherence would be associated with greater improvements on scale scores. We also hypothesized that (3) participants allocated to shorter meditations would practice more of the home meditation sessions and would be more likely to complete the study than those allocated to longer meditations.

Lastly, given the lack of evidence to suggest that the type of mindfulness meditation practice influences such adherence, we conducted an exploratory investigation to examine whether there were differences in the study dropout/completion rate based on the type of mindfulness meditation.

## Method

### Participants

As many participants as possible were recruited, and a post hoc power analysis was used to evaluate final numbers. Using G*Power (Bartlett, 2019) for the main ANOVA for the primary outcome measure (listed below) of mental well-being, the final sample size of 161 was calculated as having a statistical power of 0.75 to detect a medium effect size of Cohen’s *f* = 0.25 at a significance level of *p* < 0.05.

The study ran from January 2019 to June 2021; it was advertised through posters, a University of St Andrews weekly newsletter, social media (such as St Andrews and University of Cambridge Twitter accounts), and emails. Prospective participants were told not to participate if they self-reported experiencing low mood, having an active mental health crisis, or experiencing adverse major life events, as mindfulness meditation could increase psychological distress. If a prospective participant did not meet these (self-assessed) criteria when filling out a sign-up form, they were automatically excluded and instantly received a debrief form containing the contact information of relevant support channels. Participants were required to fulfil the following inclusion criteria of being over 18 and fluent in English, providing written consent via an online consent form.

During the data collection period, 284 participants signed up and were randomly allocated a mindfulness meditation. The average age was 32 (*SD* =  ± 14), with over 60% of participants identifying as female. The sample was primarily university-based: Over 85% were educated at undergraduate level or above. However, 115 participants dropped out of the study (did not complete the post-intervention survey), leaving 169 participants, eight further cases of which were excluded from the primary and secondary outcome data analyses due to incomplete surveys on the outcome scales, resulting in a final sample of 161 participants.

The study protocol was granted ethical approval by the St Andrews School of Psychology and Neuroscience Ethics Committee and retrospectively registered (ANZCTR12619000422123). The study was conducted online.

### Procedure

Participants who signed up were randomly allocated to one of the four mindfulness meditation interventions and asked to practice daily for 2 weeks; pre-intervention survey data were collected before the randomization—which was done via Qualtrics (evenly in blocks of one; 1:1:1:1)—otherwise participants’ arm allocation could have influenced their responses. Post-intervention (2 weeks after completing the pre-intervention survey), data were collected on the same well-being measures. The questionnaires were designed using the survey platform Qualtrics and were distributed via email. In addition, four private SoundCloud profiles were designed, each with its own meditation (Conditions 1–4) and brief description. Email reminders were also sent and participants wishing to drop out of the intervention and study could unsubscribe at any point without providing a reason. Email and survey correspondence were completed via Qualtrics, including debriefing forms being sent out.

S1 Appendix 2 provides the general descriptions for each intervention condition. Most of the meditations used were from the Oxford Mindfulness Centre’s (OMC) MBCT program and led by the same individual, Prof. Mark Williams (co-developer of MBCT and founding director of the OMC). The remaining mindful movement practice was taken from the SoundCloud of the book *Mindfulness: a practical guide to finding peace in a frantic world* (Williams & Penman, 2011). Approval to use these meditation materials was granted from Prof Williams and the current director of the OMC, Prof Willem Kuyken. Each meditation was uploaded to a separate, private SoundCloud profile and private URL links to each were provided to participants corresponding to their randomly assigned condition. Only prospective participants had access to their designated private SoundCloud profile. Since this study comprised two mindfulness practices of two different time lengths, four practices were therefore used.

Conditions 1 and 2 used a sitting meditation practice for 9.8 and 29.2 min daily, respectively (similar mindfulness practices in distinctive doses). Conditions 3 and 4 used a mindful movement practice for 8.9 and 30.1 min daily, respectively (similar mindfulness practices in distinctive doses). Similar to Yoga and Tai Chi (Clark et al., 2015), mindful movement combines mindfulness meditation with movement and stretches. In brief, it is a technique in which one endeavors to treat their present-moment experience of moving and stretching with non-reactive, unprejudiced attention, focusing awareness on the breath and/or bodily sensations with a sense of equanimity, recognizing when the mind has wandered and bringing the attention back to the breath and body. Conditions 1, 2, and 4 meditations were from the OMC’s MBCT former website/app.

### Measures

The primary outcome was the *Warwick-Edinburgh Mental Well-Being Scale*—WEMWBS (Tennant et al., 2007). This consists of 14 items measured on a 5-point Likert scale. For example, Item 1 is “I’ve been feeling optimistic about the future” with the responses ranging from *none of the time* to *all of the time*. The WEMWBS is appropriate for adult samples comprising over 100 respondents, including positively keyed items for assessing mental well-being, with higher scores indicative of greater well-being (Tennant et al., 2007). Permission was attained to use this.

*General Population-Clinical Outcomes in Routine Evaluation Scale*—GP-CORE (Evans et al., 2005) was a secondary outcome. The freely available 14-item, 5-point Likert scale is a measure of general psychological distress in non-clinical samples (Evans et al., 2005). Lower scores indicate less distress. The GP-CORE is appropriate for general adult populations, containing low risk and intensity items, more than half of which are keyed positively to augment its suitability across non-clinical samples (Evans et al., 2005). For example, item 14 is “I have achieved the things I wanted to” with responses ranging from *not at all* to *most or all of the time*.

The other secondary outcome was the Mindfulness Attention Awareness Scale (MAAS; Brown & Ryan, 2003). The freely available 15-item, 6-point Likert scale measures the core distinctive properties of dispositional mindfulness, specifically open alertness of and attention to what is happening in the present and has been validated with university and general public samples (Brown & Ryan, 2003). MAAS includes negative items only; for instance, Item 7 is “It seems I am ‘running on automatic,’ without much awareness of what I’m doing” with responses ranging from *almost always* to *almost never*. Higher scores denote greater tendency towards mindfulness, while lower scores denote a tendency towards mindlessness (Brown & Ryan, 2003). The items are reversed scored since they all inquire about mindlessness. Since MAAS does not directly measure mindfulness, but instead mindlessness, this is raised as a limitation in the “Discussion” section.

Adherence to recommended daily practice was simply collected by asking participants how many sessions they practiced (out of the 14 home meditation sessions assigned). Seeing as this was a retrospective self-report, the limitations of self-reported data are outlined in the “Discussion” section. Study completion/dropout rate was measured by observing how many people dropped out after being randomly assigned to a mindfulness meditation intervention. In other words, dropout was evaluated by post-intervention questionnaire completion.

### Data Analyses

Participant data were collected online through Qualtrics and stored in an anonymized format. IBM SPSS Statistics version 24 on MacOS was used to analyze the data, with the significance level throughout set as *p* < 0.05.

Time (pre vs. post) was the factor of interest for the main analysis. We started with a three-way (type by dose by time) ANOVA to find an effect on the separate measures of mental well-being, psychological distress, and dispositional mindfulness, with any significant effects being followed up with *t*-tests. This analysis was then rerun as a two-way (type by dose) ANOVA on the difference scores (on the primary and secondary outcome measures), reporting the lack of any significant effects. A per-protocol analysis was used since our main aim was to detect differences between doses. Cronbach’s alphas (*α*) and McDonald’s omegas (*ω*) were used to examine the internal reliability of the primary and secondary outcome scales. ANCOVA with one covariate (self-reported adherence) was then run to analyze whether self-reported adherence to recommended daily practice influenced the difference between pre-post intervention scores on the outcome scales, with any significant results followed up by a two-way ANOVA.

Lastly, a binary logistic regression was run to examine the dropout rate of participants depending on the condition they were randomly assigned to, along with a follow-up chi-square test of any significant results to examine the association between such categorical variables.

## Results

**Fig. 1:**
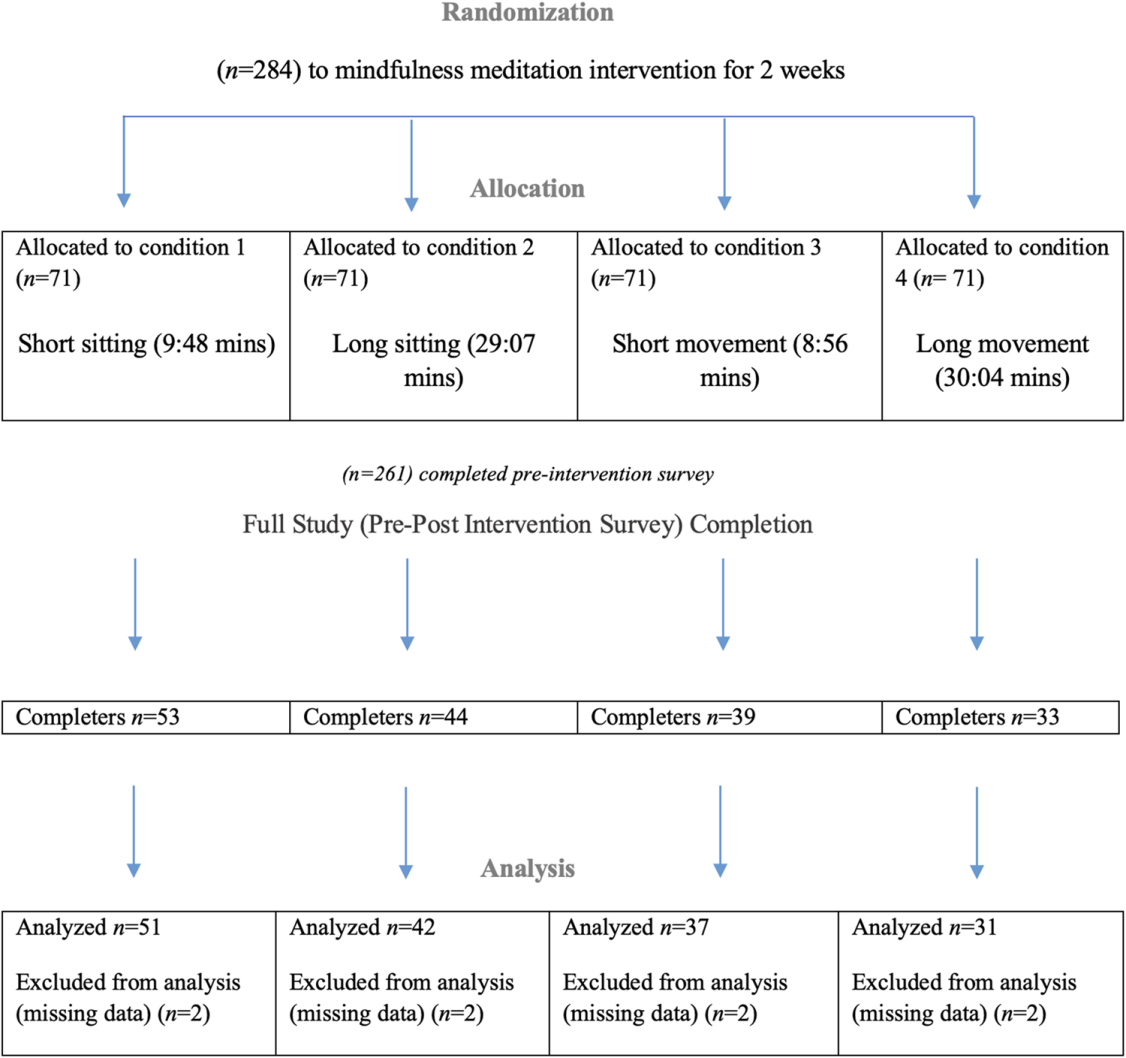
Study flowchart

**Fig. 2:**
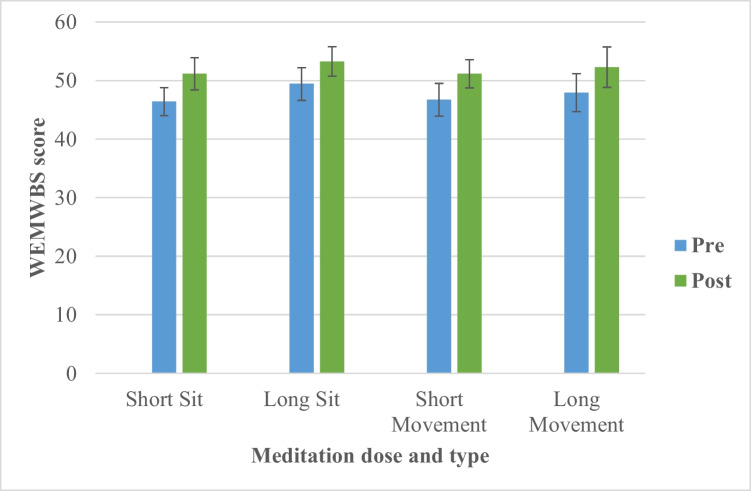
Mean (± 95% CI) WEMWBS scores for the four mindfulness meditation conditions. Blue bars, pre-intervention scores; green bars, post-intervention scores; possible score range, 14–70; higher scores are indicative of greater well-being

**Fig. 3:**
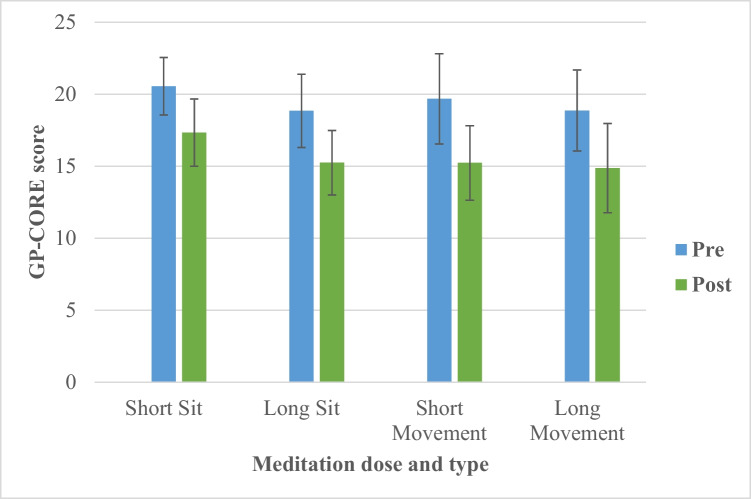
Mean (± 95% CI) GP-CORE scores for the four mindfulness meditation conditions. Blue bars, pre-intervention scores; green bars, post-intervention scores; possible score range, 0–56; lower scores are indicative of less distress

**Fig. 4:**
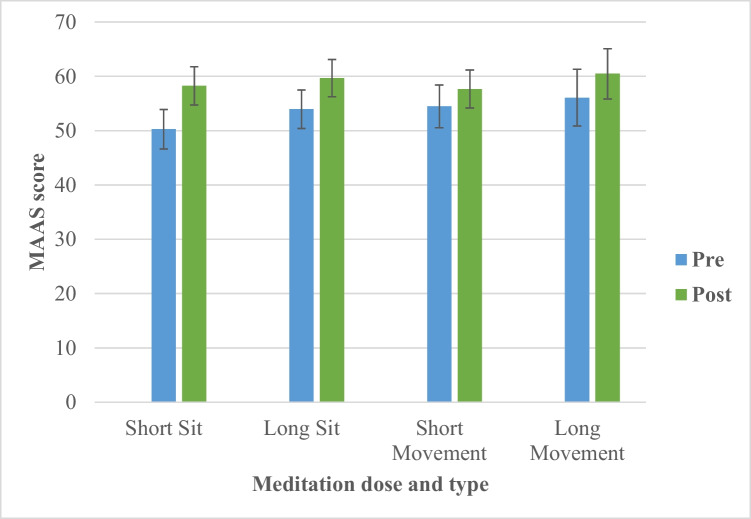
Mean (± 95% CI) MAAS scores for the four mindfulness meditation conditions. Blue bars, pre-intervention scores; green bars, post-intervention scores; possible score range, 15–90; higher scores are indicative of greater mindfulness

Table 1 displays the demographic information along with baseline scores on the primary and secondary outcomes. No significant differences were found among the four conditions, as per one-way ANOVAs across the conditions for the continuous data, and chi-square tests for the categorical variables. Figure 1 presents the study flowchart and Figs. 2, 3, and 4 present the pre-intervention (baseline) and post-intervention scores by condition on the primary and secondary outcomes.

**Table 1:** Demographic sample characteristics and mean (± standard deviation) baseline scores

		Condition			
Characteristic	One (*n* = 51)	Two (*n* = 42)	Three (*n* = 37)	Four (*n* = 31)	*p*
Female (*n*)	37 (73%)	28 (67%)	24 (65%)	20 (65%)	0.838
Age (± SD)	31.7 ± 15	29.6 ± 12.4	29.5 ± 12.7	33.7 ± 12.7	0.512
Education (*n*)					
Postgraduate Undergraduate	16 (31%) 26 (51%)	16 (38%) 23 (55%)	16 (43%) 18 (49%)	14 (45%) 13 (42%)	0.599
WEMWBS GP-CORE MAAS	46.4 ± 8.51 20.6 ± 7.12 50.3 ± 12.9	49.4 ± 9 18.8 ± 8.2 54 ± 11.4	46.7 ± 8.40 19.7 ± 9.42 54.5 ± 11.8	47.9 ± 8.90 18.9 ± 7.70 56.1 ± 14.3	0.360 0.721 0.182

%, percentage within condition

### Mental Well-being

Both pre-intervention WEMWBS (14 items; *α* = 0.906, *ω* = 0.905) and post-intervention WEMWBS (14 items; *α* = 0.932, *ω* = 0.932) were found to be very highly reliable. There was a significant main effect of time (pre-post intervention) on WEMWBS scores (*F* (1,157) = 54.4, *p* < 0.001, *η*2 = 0.257). However, there was no significant main effect of either meditation type or dose, nor a significant interaction between type and dose between the conditions on pre-post WEMWBS scores. Furthermore, there were no significant interactions between either type of meditation or dose and pre-post WEMWBS scores, nor was there a significant interaction between type, dose, and pre-post WEMWBS difference scores.

The significant main effect of time was followed up with paired *t*-tests. As shown in Fig. 2, results displayed that pre-intervention WEMWBS scores significantly increased post-intervention for all conditions: short/sitting (*t*(50) = 3.78, *p* < 0.001, *d* = 0.528), long/sitting (*t*(41) = 3.76, *p* = 0.001, *d* = 0.580), short/movement (*t*(36) = 4.23, *p* < 0.001, *d* = 0.695), and long/movement (*t*(30) = 3.87, *p* = 0.001, *d* = 0.696).

### Psychological Distress

Both pre-intervention GP-CORE (14 items; *α* = 0.742, *ω* = 0.727) and post-intervention GP-CORE (14 items; *α* = 0.807, *ω* = 0.805) were found to be reliable. There was a significant main effect of time on GP-CORE scores (*F* (1,157) = 46.7, *p* < 0.001, *η*2 = 0.229). However, there were no significant main effects of either meditation type or dose, nor a significant interaction between type and dose between the conditions on pre-post GP-CORE scores. Furthermore, there were no significant interactions between the type of meditation, nor the dose and pre-post GP-CORE scores, nor was there a significant interaction between type, dose, and pre-post GP-CORE difference scores.

As shown in Fig. 3, pre-intervention GP-CORE scores significantly decreased post-intervention for all four conditions: short/sitting (*t*(50) =  − 3.02, *p* = 0.004, *d* =  − 0.422), long/sitting (*t*(41) =  − 3.88, *p* < 0.001, *d* =  − 0.598), short/movement (*t*(36) =  − 3.62, *p* = 0.001, *d* =  − 0.596), and long/movement (*t*(30) =  − 3.47, *p* = 0.002, *d* =  − 0.623). However, moderately high standard deviations (*SD*s) relative to the mean (*M*) scores suggest substantial variance in the data.

### Dispositional Mindfulness

Both pre-intervention MAAS (14 items; *α* = 0.883, *ω* = 0.882) and post-intervention MAAS (14 items; *α* = 0.889, *ω* = 0.889) were found to be highly reliable.

There was a significant main effect of time on MAAS scores (*F* (1,157) = 43.8, *p* < 0.001, *η*2 = 0.218). However, there was no significant main effect of either meditation type or dose, nor a significant interaction between type and dose between the conditions on pre-post MAAS scores. Furthermore, there were no significant interactions between the type of meditation, nor the dose and pre-post MAAS scores, nor was there a significant interaction between type, dose, and pre-post MAAS difference scores.

As shown in Fig. 4, pre-intervention MAAS scores significantly increased post-intervention for three conditions: short/sitting (*t*(50) = 5.24, *p* < 0.001, *d* = 0.733), long/sitting (*t*(41) = 4.20, *p* < 0.001, *d* = 0.648), and long/movement (*t*(30) = 2.33, *p* = 0.027, *d* = 0.419). MAAS scores for the remaining condition increased and approached significance: short/movement (*t*(36) = 2.00, *p* = 0.054, *d* = 0.328).

### Correlations

There were significant correlations between the changes on the primary outcome WEMWBS and changes in the secondary outcomes GP-CORE (*r*(158) =  − 0.700, *p* < 0.001) and between WEMWBS and MAAS (*r*(158) = 0.467, *p* < 0.001). There was also a significant correlation between changes on secondary outcomes GP-CORE and MAAS (*r*(158) =  − 0.478, *p* < 0.001). When controlling for changes on MAAS, the correlation between changes on WEMWBS and GP-CORE remained significant and strong (*r*(158) =  − 0.613, *p* < 0.001). The lack of differences between conditions may be due to a suboptimal sample size (actual study power was 75%), although this is unlikely given that the effect sizes were so close to the null for well-being (*η*2 = 0.001), distress (*η*2 = 0.001), and dispositional mindfulness (*η*2 = 0.007). Moreover, on visual inspection of Figs. 2, 3, and 4 for our main analyses, there did not appear to be any obvious difference between conditions pertaining to change in primary and secondary outcome measure scores.

### Self-Reported Adherence

A two-way (type by dose) ANCOVA with one covariate (self-reported adherence) was then run to analyze whether self-reported adherence to recommended daily practice influenced the difference between pre-post intervention scores on the three outcome scales. Even after adding the covariate, there was still no significant main effect of either meditation type or dose, nor interaction between these factors, on any of the pre-post scale scores. This was most likely due to the extremely small effect sizes observed here (Table 2).

**Table 2:** Correlations between self-reported adherence and pre-post scale score differences

Pre-post score difference	Self-reported adherence
WEMWBS	0.186*
GP-CORE	− 0.192*
MAAS	0.215**

^*^Correlation is significant at the *p* < 0.05 level (two-tailed); **correlation is significant at the *p* < 0.01 level (two-tailed), *n* = 161

Albeit with weak effect sizes, more regular practice across all conditions was associated with a significant increase in WEMWBS and MAAS scores along with a significant decrease in GP-CORE scores.

A two-way ANOVA was then used to compare participants’ self-reported adherence between short- and long-dose mindfulness meditations along with the type of meditation (sitting and movement). Although the average self-reported adherence was slightly higher for the shorter meditations (*M* = 8.43 sessions, *SD* = 3.71) than the longer meditations (*M* = 7.55 sessions, *SD* = 3.78), the difference was not significant (*F* (1,157) = 1.89, *p* = 0.171, *η*2 = 0.012). Moreover, the results showed that there was no significant main effect of meditation type on self-reported adherence (*F* (1,157) = 0.652, *p* = 0.421, *η*2 = 0.004) and no significant interaction between meditation type and dose (*F* (1,157) = 0.259, *p* = 0.611, *η*2 = 0.002).

### Study Completion and Dropout Rate

Binary logistic regression was used to assess the dropout rate, defined as not completing the post-intervention survey, for all participants who were allocated a meditation based on the type and dose of mindfulness practice they were randomly assigned to.

As per Table 3, meditation type was a statistically significant predictor variable, whereas meditation dose was non-significant. The odds of dropping out of the study were 2.54 times higher for participants assigned a movement meditation than a sitting meditation. However, because this result was not expected, it should be interpreted with caution.

**Table 3:** Summary of binary logistic regression for expected probabilities of a participant dropping out of the study for the two predictors

	*B*	*SE*	*Wald*	*df*	*p*	*Odds ratio* [95% CI]
Meditation type	0.933	0.250	13.9	1	< 0.001	2.54 [1.56, 4.15]
Meditation dose	0.401	0.250	2.59	1	0.108	1.49 [0.916, 2.44]

*B* logistic regression coefficient, *SE* std. error of coefficient, *df* degrees of freedom, *CI* confidence intervals

A chi-square test followed up the significant result. There was a significant association between meditation type and whether a participant dropped out of the study, *χ*^2^_1_ = 14, *p* < 0.001, *φ* = 0.222. A total of 97 participants allocated to sitting meditations completed the study, which is significantly higher than the 72 participants allocated to movement meditations. Among participants who dropped out after being allocated to a mindfulness practice, 70 were assigned to movement meditations, which is significantly higher than the 45 allocated to sitting meditations.

We also examined baseline differences on the outcome measures for participants who dropped out but fully completed the pre-intervention survey (*n* = 100) in comparison to those who fully completed the entire study, including both pre- and post-intervention surveys (*n* = 161). No significant differences were found between the two groups (participants who dropped out vs. participants who fully completed the study) in terms of scores on WEMWBS (*F*(1, 259) = 0.011, *p* = 0.915, *η*2 < 0.001), GP-CORE (*F*(1, 259) = 0.241, *p* = 0.624, *η*2 = 0.001), nor MAAS (*F*(1, 259) = 0.115, *p* = 0.735, *η*2 < 0.001).

## Discussion

The main results of this trial were that, while self-reported significant improvements in mental well-being, psychological distress, and dispositional mindfulness were found regardless of mindfulness meditation type and dose, no significant differences concerning effects between the conditions were detected. We believe this lack of difference between conditions paired with significant improvements within each condition yields intriguing insights.

There are still very few component analyses in the mindfulness meditation field; this work builds on previous literature on the topic of meditation type, but also extends it by factoring in the durations of meditation. Recently, one study aimed to disentangle the effects of three mindfulness meditations (observing thoughts, body scan, and mindful breathing) on rumination and time perspective in a university sample (*n* = 75). Feruglio et al. (2021) found a specific effect of the breathing mindfulness meditation in reducing and increasing mental rumination and optimistic outlook, respectively, in comparison to all other conditions. Another recent mindfulness-based component analysis in another non-clinical student sample (*n* = 56) examined mindful breathing, the body scan, and loving-kindness meditation (Kropp & Sedlmeier, 2019). In comparison to the mindful breathing and loving-kindness meditations, the authors detected an effect of the body scan on life satisfaction, emotion regulation, and self-compassion. Perhaps the most similar component analysis study to ours (Sauer-Zavala et al., 2013) examined mindful sitting meditation, mindful Yoga (movement), and the body scan, in another university student sample (*n* = 141). Contrary to our findings, mindful movement was associated with larger improvements in well-being than the sitting meditation (and body scan).

The above study periods were 8 weeks (Feruglio et al., 2021), 6 weeks (Kropp & Sedlmeier, 2019), and 3 weeks (Sauer-Zavala et al., 2013). Accordingly, our study period of 2 weeks may not have been long enough to detect significant differences across different types of mindfulness meditation. Though limited research remains on this topic, these collective findings set the scene for future research to explore differential effects of different meditative techniques, informing design and (re)structuring of MBSH programs along with better matching of specific techniques to specific populations, so that practitioners can derive most benefit.

The mindfulness meditation interventions were associated with increases in mental well-being and decreases in psychological distress. These findings provide preliminary evidence for the effectiveness of fully automated mindfulness interventions (Davis & Zautra, 2013; Mak et al., 2015; Morledge et al., 2013). However, again, there were no significant differences on the mental well-being scores and psychological distress scores across conditions. Another practical explanation is that, like certain exercise routines, specific meditation techniques and doses work for different people (Goleman & Davidson, 2017). Further, our findings suggest that one mindfulness meditation component type (sitting vs. movement) or dose (short vs. long) is not necessarily superior to the other with regard to improving mental well-being.

Furthermore, our online study also tentatively provides support for the finding that mindfulness meditation can be effective when delivered remotely (Gu et al., 2018; Spijkerman et al., 2016). Nonetheless, some populations, particularly those which are vulnerable may struggle with meditation interventions (Przyrembel et al., 2019) and first doing-no-harm should always take center stage between clinicians and their patients.

Another finding was that all the mindfulness meditations were associated with increases in dispositional mindfulness (or decreases in mindlessness) but there were no significant differences on the mindfulness scores between conditions based on meditation type and dose. Kropp and Sedlmeier (2019), on the other hand, found greater increases in mindfulness for a body scan condition than mindful movement. Our finding is surprising since it would be expected that greater meditation doses would be associated with increases on mindfulness scores. While increased meditation dose is connected to higher levels of dispositional mindfulness (Brown & Ryan, 2003), longer meditation doses were not correlated with significantly greater increases in dispositional mindfulness in comparison to shorter meditations in this study. Longer studies paired with larger sample sizes may be able to tease out the differences, if any, between conditions more effectively. Moreover, expectancy and placebo effects may provide explanation for why dose and type do not seem to matter in the case of this work. Thus, we suggest future studies concerned with this should include measures such as the credibility and expectancy questionnaire (CEQ-6) (Devilly & Borkovec, 2000) to see if significant differences between groups are present regarding the mindfulness meditation intervention randomly allocated to participants.

Adherence data involved retrospective self-report, introducing significant limitations (mentioned below) which means any conclusions on this would be tentative at best. Shorter MBCT meditations were not significantly practiced more regularly than the longer MBCT meditations. Hence, after assessing the self-reported rate of daily practice over 2 weeks, the results did not support the widely held belief that lower doses of meditation are easier to practice. Again, this finding is interesting as it does not align with the common sensical and logical assumption that lower meditation duration is inevitably easier to do. It contradicts previous findings of dose mediating engagement in mindfulness-based programs (Banerjee et al., 2017). An alternative explanation is that, like the techniques/types of meditation components, certain doses may work best for different individuals. Additionally, there were no significant differences in the self-reported adherence for mindfulness sitting meditations and movement meditations. There was no prior evidence in the literature to suggest that this explorative inquiry would result differently. Self-reported adherence was still low since the meditations were only practiced ~ 7–8 days on average across the 2 weeks (half the recommended daily practice), corresponding with compliance rates in the vast literature on mindfulness meditation and self-help interventions in general (Banerjee et al., 2017).

Interestingly, there was a significant difference in dropout rate depending on whether participants were allocated to mindfulness sitting meditations or movement meditations. Participants practicing mindful movement dropped out significantly more, irrespective of dose, which suggests that this technique may be harder to adhere to. However, this result could be spurious due to the use of multiple statistical tests, so follow-up studies are needed to ascertain the relationship between adherence and well-being outcomes. Given the unexpected nature of this result, it should be treated as a tentative finding. Alternatively, it should be considered that the movement meditation chosen for this study was simply not effective at improving well-being, and the main reason it appeared to be as effective as the sitting meditation was that most of the participants for whom it was not working well did not complete the study. Moreover, while sitting meditations attending to one’s breathing are relatively well-defined—and are therefore more likely to be similar across studies—there is greater potential for variation in how movement meditations are conducted (i.e., Yoga vs. Tai Chi), making it more difficult to generalize from the mindful movement practice used in our study.

### Limitations and Future Directions

Our study contains several limitations. Well-being could have improved because of unspecific effects—for example, simply due to participants dedicating some time to their mental and/or physical health, or even regression to the mean (common in pre-post results). Participants practiced the mindfulness meditations during distinctive seasons, which can profoundly impact individuals’ well-being (Rosenthal et al., 1984) and, in turn, subjective measures of well-being. This is a common phenomenon in longitudinal meditation research (Kok & Singer, 2017), and may be corrected by having all participants complete interventions at the same time along with the respective measures, self-report or otherwise. This should not understate the lengthy period of our study and is further confounded by the fact there was no attempt to balance the four conditions continuously over this period, introducing more potential bias.

Another common drawback in meditation research that applied to this study was the high attrition rate (Galante et al., 2014). The total dropout rate of this study was high at 40.5%, conforming with the literature on attrition in online and automated mindfulness meditation and self-help interventions in general (Banerjee et al., 2017; Galante et al., 2016; Mak et al., 2015; Morledge et al., 2013). For instance, a meta-analysis on MBSH interventions reported a dropout rate of 37% (Cavanagh et al., 2014). Future research needs to investigate factors that influence study completion and dropout rate, with the aim of developing novel and nuanced ways to increase participation/adherence in MBSH. Nonetheless, the generalizability of our results may be hampered by the large difference in dropout rates between the two types of meditation. This is compounded by the fact that movement meditations in particular can vary a great deal relative to sitting meditations focused on attention to breathing.

A further limitation was the sizeable variation in the data, and the unequal sample sizes across the conditions could have affected the already suboptimal statistical power in detecting differences. Furthermore, like similar component analyses, the sample in the current study was primarily drawn from a university population, limiting the generalizability of the findings further. Future research should aim to study more diverse samples in comparison to the archetypal population (Henrich et al., 2010) found here (along with many other studies), so as to provide greater balance.

Perhaps one of the biggest limitations is that, due to time and resource constraints, we did not actively report on and measure adverse effects. The many participants who did not fully complete this study could have experienced adverse effects, or at least not have perceived any benefits. Likewise, it is crucial to note that we only examined two mindfulness practices, movement meditation and siting meditation. Future research should compare other techniques such as “Stretch and Breathe” (used in MBCT), which is a combination of the sitting practice and mindful movement used in the present study. It could be feasible to predict that this sitting and movement combination may impact well-being differently to its separate components in isolation. As shown in similar studies, other outcomes such as self-compassion and rumination may be affected. Such component analyses offer a chance to match the best practices to desired outcomes, along with merging multiple practices into one (for example, combining a sequence of standing mindful movement, breathing, sitting, and lying body scan with loving-kindness meditation).

Another serious concern is the quality of the adherence data, which was self-reported retrospectively. Accordingly, these data should be viewed cautiously. We do not know whether participants recollected their practices accurately. Future mindfulness research which is conducted remotely or online could better validate this by attempting to objectively track adherence in nuanced ways. Moreover, although the meditations were led by the same instructor, there were differences in the instructions, possibly adding a confounding condition. Equally, though MAAS was the best available outcome measure we had at the time, this measurement of dispositional mindfulness may also be flawed since it focused on items pertaining to mindlessness.

Lastly, direct comparisons should be made between online and live delivery (along with retrospectively and in real-time) of these meditations to understand differences further, and future mindfulness-based component analyses could include both meditation type and dose. Just as there is a 17-item MBCT Adherence Scale (MBCT-AS) to examine therapists delivering this meditation program and their adherence to the MBCT protocol (Segal et al., 2002), an adherence scale could also be developed for participants involved in mindfulness-based programs. Data collected from this conceptual scale from retrospective populations and those currently (and in the future) completing courses involving mindfulness, with specific feedback on the meditation components (and how often they were practiced), could help increase adherence (to recommended practice and the course as a whole) in future MBSH and mindfulness-based programs in general.

## Supplementary Information

Below is the link to the electronic supplementary material.

Supplementary file 1 (DOCX 1348 KB)

Supplementary file 2 (DOC 219 KB)

## Data Availability

Data will be made available upon publication in a public repository.
