# Supplementary material for: Effects of Mindfulness Meditation Duration and Type on Well-being: an Online Dose-Ranging Randomized Controlled Trial
Source: Mindfulness (N Y). 2023 Apr 12;14(5):1171–82. doi: 10.1007/s12671-023-02119-2 (PMC10090715; doi:10.1007/s12671-023-02119-2)
Supplement: Supplementary file 1 — (DOCX 1348 KB) [file 12671_2023_2119_MOESM1_ESM.docx]

**Supplementary Material**

**Effects of mindfulness meditation duration and type on well-being: An online dose-ranging randomized-controlled trial**

**S1 Appendix 1—Mindfulness meditation material**


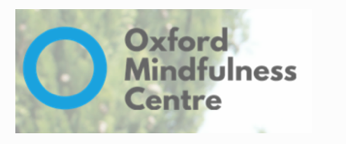


**
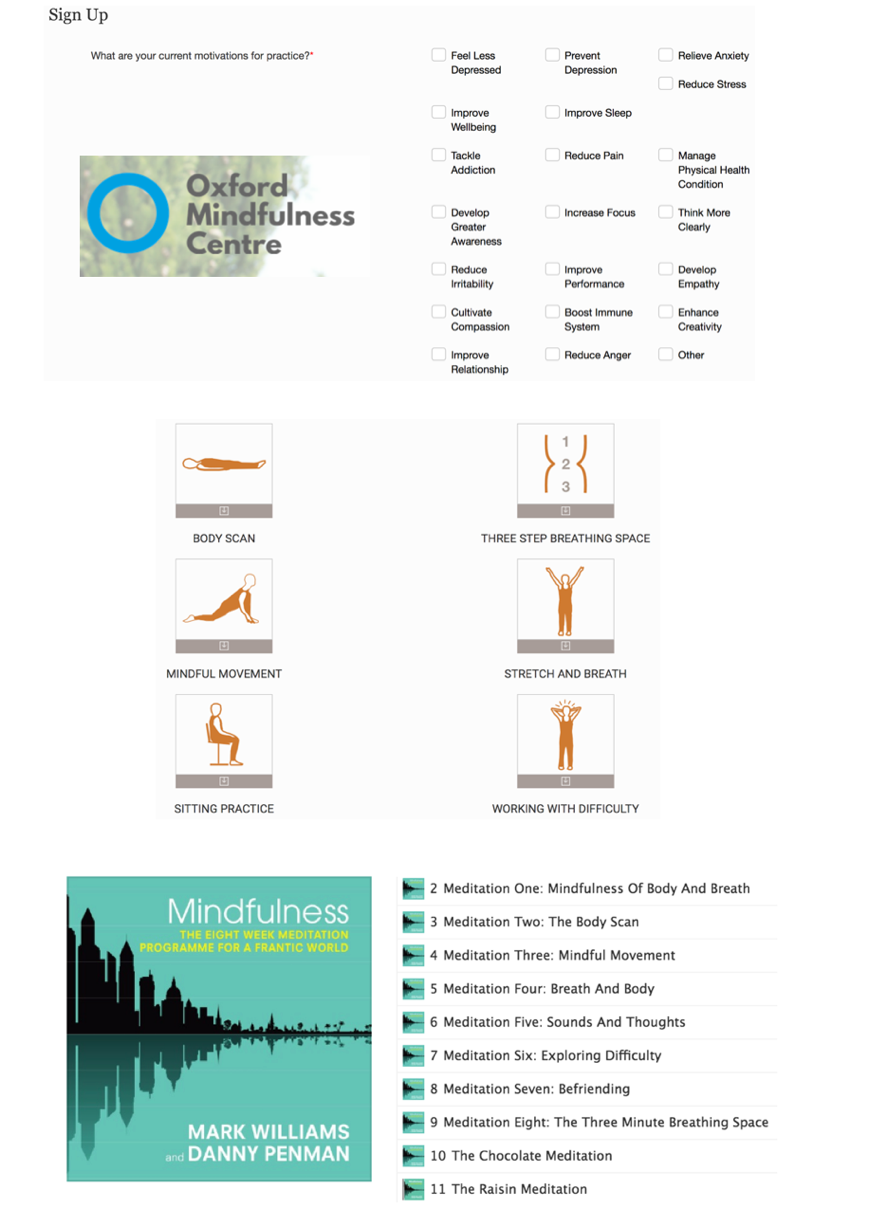
**

*From: Oxford Mindfulness Centre and Finding Peace in a Frantic World book*

**S1 Appendix 2—Secret SoundCloud meditations**

**
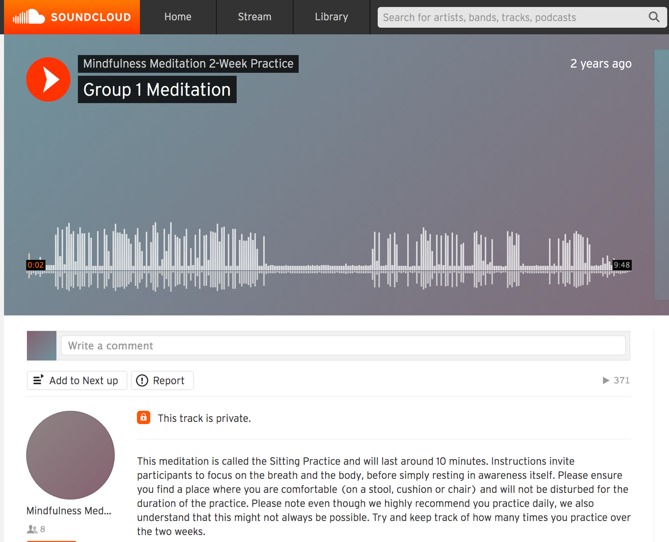

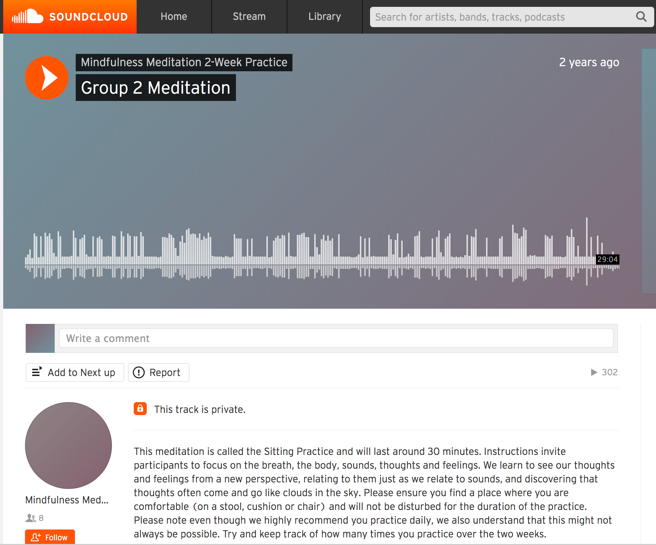
**

**
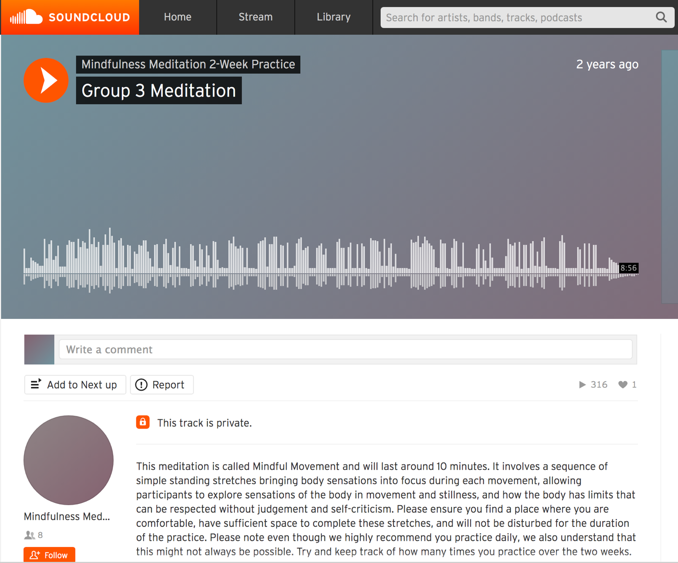

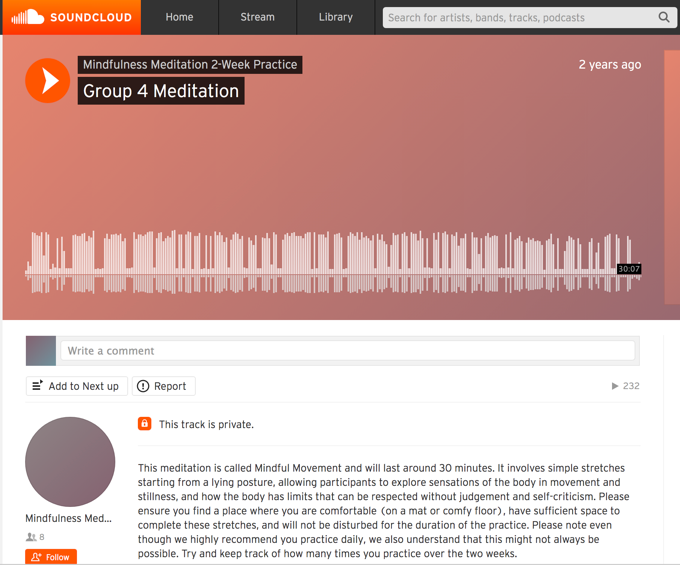
**

[**Short sitting**](https://soundcloud.com/mbct-two-week-study/group-1-meditation/s-6rGwI?si=ecfd2d6b84af4e2fbd201a798439e699&utm_source=clipboard&utm_medium=text&utm_campaign=social_sharing) [**Long sitting**](https://soundcloud.com/mbct-two-week-study/group-2-meditation/s-CB1LM?si=ecfd2d6b84af4e2fbd201a798439e699&utm_source=clipboard&utm_medium=text&utm_campaign=social_sharing)

[**Short movement**](https://soundcloud.com/mbct-two-week-study/group-3-meditation/s-Qa1OG?si=ecfd2d6b84af4e2fbd201a798439e699&utm_source=clipboard&utm_medium=text&utm_campaign=social_sharing) [**Long movement**](https://soundcloud.com/mbct-two-week-study/group-4-meditation/s-byl4f?si=ecfd2d6b84af4e2fbd201a798439e699&utm_source=clipboard&utm_medium=text&utm_campaign=social_sharing)
